# The potential role of miR-155 in the tumorigenesis of cervical cancer

**DOI:** 10.3389/fcell.2025.1572373

**Published:** 2025-10-03

**Authors:** Xu Chen, Liying Zheng, Xianxu Zeng, Nanying Pan

**Affiliations:** ^1^ Department of Pathology, Third Affiliated Hospital of Zhengzhou University, Zhengzhou, Henan, China; ^2^ Postgraduate Department, First Affiliated Hospital of Gannan Medical College, Ganzhou, Jiangxi, China; ^3^ Department of Obstetrics and Gynecology, Taizhou Central Hospital (Taizhou University Hospital), Taizhou, China

**Keywords:** cervical cancer, tumorigenesis, prognosis, mechanism, clnical

## Abstract

Cervical cancer poses a major threat to the health of females and is the leading cause of mortality in females. Despite advancements in treatment, most cervical cancer patients have a poor prognosis due to late diagnosis and resistance to treatment. Investigating microRNAs introduces a new path for developing cancer prevention and treatment. miR-155, one of many microRNAs, plays an essential role in tumor development by regulating gene expression processes, such as transcription, translation, and splicing. In cervical cancer, a number of studies have been conducted to exploring the role and mechanisms of miR-155. Therefore, a comprehensive review summarizing all available findings is necessary to clarify the role of miR-155 in cervical cancer development and progression. This review presents an overview of the state-of-the-art research in miR-155 for cervical cancer, including cell apoptosis, migration, invasion, and drug resistance, and highlights its potential as a biomarker and therapeutic target for cervical cancer treatment and prognosis.

## Introduction

As per GLOBOCAN2020, there are 604,127 new cases of cervical cancer annually, comprising 6.5 percent of all malignant tumors in women worldwide (Sung et al., 2021). Cervical cancer is the fourth most common and lethal neoplasms among women worldwide (Sung et al., 2021). In addition, there were approximately 341,831 deaths due to cervical cancer every year (Sung et al., 2021). Cervical cancer represents a significant global health burden, particularly in developing countries where access to screening and treatment is limited. Continuous infection of high-risk human papillomavirus (HPV) types is closely associated with cervical cancer (Perkins et al., 2023). In most cases (90%), HPV infections are cleared within 6–18 months by the immune system (Fraszczak et al., 2022). However, the high-risk subtypes of HPV infection sometimes persists, such as HPV16 and HPV18, and express two viral oncogenes E6 and E7 (Singini et al., 2023). Subsequently, the E6 and E7 oncogenes allow the production of oncoproteins E6 and E7 through transcription or translation, which lead to increased genomic instability and accumulation of somatic mutations resulting in cervical cancer (Organista-Nava et al., 2019). The uterine cervix present multiple pathophysiological conditions, including cervicitis, cervical polyps, cervical warts, pelvic inflammatory disease (PID), cervical dysplasia, and cervical cancer. The progression of persistent HPV infection cells into cervical warts, cervical intraepithelial neoplasia and finally into invasive invasive cancer (Organista-Nava et al., 2019).

Despite diagnosis and treatment advances in recent years, the morbidity and mortality rate of cervical cancer remain high, especially in the advanced stage. It is widely acknowledged that radical surgery or radiotherapy are the preferred treatment for most of patients with early-stage cervical cancer. While chemotherapy and neoadjuvant chemotherapy are the basic treatment options for patients with advanced cervical cancer and these patients have a poor prognosis due to resistance to chemotherapy. Therefore, an in-depth study on the underlying molecular mechanisms of metastasis and resistance to chemotherapy in cervical cancer would be of great significance for its prevention, early diagnosis and treatment.

In recent years, with the profound study of microRNAs (miRNAs), new horizons for the prevention diagnosis and treatment of cancers are opened (Farooqi et al., 2022). miRNAs have evolved considerably since it was discovered. miRNAs have been reported to widely involved in cell proliferation, cell cycle, differentiation, apoptosis regulation processes (Louro et al., 2022; Shahbazi-Derakhshi et al., 2023; Wang et al., 2023). Recently, numerous studies have indicated that miRNAs potentially played important roles in tumorigenesis, including cervical cancer (Salum et al., 2024a; Li et al., 2024; Srinath et al., 2024). One of the most studied miRNAs in cancers is miR-155, which participated in the development and progression of various cancers, including cervical cancer (Alexandre et al., 2024; Shen et al., 2024; Wang et al., 2016), breast cancer (Grimaldi et al., 2020), oral cancer (Maheswari et al., 2024), gastric cancer (Su et al., 2022), liver cancer (Wang et al., 2021), and lung cancer (Fang et al., 2024). Furthermore, while some studies have implicated miR-155 dysregulation in cervical cancer, conflicting findings and limited data hampered the progress for a comprehensive understanding of disease pathogenesis. Additionally, miR-155 is recognized as a diagnostic, therapeutic and prognostic target in multiple cancers (Karajovic et al., 2024; Wang et al., 2024). Therefore, a deeper understanding of the role of miR-155 in cervical cancer may facilitate the development of targeted therapies tailored to individual patients, ultimately improving treatment outcomes and reducing the burden of this devastating disease. In this review, we summarize and discuss the potential role of miR-155 as a new biomarker or therapeutic target in cervical cancer.

### Overview of miR-155

miRNAs, the most frequent type of non-coding RNA, consist of 19–25 nucleotides, and repress and degrade the translation of target mRNA by binding to the 3’untranslated region (UTR) (Guo et al., 2022; Liu et al., 2020). However, in rare cases, miRNAs promote target genes translation and is involved in post-transcriptional gene regulation (Ha and Kim, 2014). At present, a total of 1,572 miRNAs have been identified and about one-third of all human genes are negatively targeted and regulated by miRNAs at transcriptional or post-transcriptional levels (Song et al., 2023; Zhao et al., 2022). The biogenesis of miRNAs involves a series of coordinated steps, beginning in the nucleus and culminating in the cytoplasm. The majority of miRNAs are transcribed by RNA polymerase II as long primary miRNA transcripts (pri-miRNAs) from genomic DNA (Bhat et al., 2020). Pri-miRNAs can be several kilobases in length and often contain multiple hairpin structures, each capable of producing a mature miRNA (Li N. et al., 2022). The microprocessor complex, consisting of the RNA-binding protein DGCR8 and the endonuclease Drosha, removes the5′ and 3′ends of the pri-miRNA (Sun et al., 2022). This cleavage event occurs near the base of the hairpin, resulting in the release of a hairpin-shaped precursor miRNA (pre-miRNA) approximately 70–100 nucleotides in length (Sun et al., 2022). The pre-miRNA is recognized by Exportin-5, a member of the Ran-GTP-dependent nuclear export receptor family, and then is exported from the nucleus (Cambiagno et al., 2021). The pre-miRNA is further processed by the RNase III enzyme Dicer, along with its co-factor TRBP (HIV-1 TAR RNA-binding protein), to generate a mature miRNA duplex (Ma S. et al., 2023). The mature miRNA duplex is loaded onto the RNA-induced silencing complex, where one strand (the guide strand) is preferentially selected for incorporation based on thermodynamic stability and sequence composition (Kim et al., 2019). MiRNAs primarily bind to the 3′untranslated region (UTR) of target mRNAs through sequence complementarity, typically leading to translational repression or mRNA degradation (Ma J. et al., 2023). Overall, the biogenesis of miRNAs is a highly regulated and complex process that involves multiple protein complexes and cellular compartments (El-Meguid et al., 2024; Salum et al., 2024a). Dysregulation of miRNA biogenesis can have profound effects on gene expression and cellular function, contributing to various diseases, including cancer, neurodegenerative disorders, and cardiovascular diseases.

miR-155, a small non-coding RNA molecule, is encoded by the miR-155 host gene located on chromosome 21 in humans (Yang W. et al., 2022). Generally, mature sequences of miR-155 is divided into 3p and 5p according the source, miR-155-5p occupies a dominant position in miR-155 (Han et al., 2022). It is highly conserved across species and is expressed in a variety of cell types, including immune cells, epithelial cells, and cancer cells (Asadirad et al., 2022; Ji et al., 2022; Li X. et al., 2022). miR-155 plays diverse roles in physiological processes and pathological conditions, including apoptosis, proliferation and migration, owing to its ability to regulate the expression of target genes post-transcriptionally (Kalkusova et al., 2022). Also, miR-155 is significantly differentially expressed in immune regulation, metabolic regulation, inflammation, neurological disorders and various tumors (Yao et al., 2020; Chen et al., 2021; Gulei et al., 2019; Ke et al., 2023; Rastegar-Moghaddam et al., 2023; Zhang et al., 2022). For example, Wen et al. (Wen et al., 2021) showed that SIRT1, regulating inflammation, metabolism and other physiological processes, was a miR-155 downstream target gene and that inhibition of miR-155-5p alleviates neuroinflammation through the activation of SIRT1 in the trigeminal nucleus caudalis of chronic migraine mice. In addition, miR-155 was reported to have tumor-suppressive or tumor-promoting consequences. Qin et al. (2013) found that CLDN1 was a well-known gene for ovarian cancer cell invasion and adhesion, and miR-155 significantly suppressed the proliferative and invasive capacity of ovarian cancer cell and inhibited the growth of ovarian xenograft tumors by downregulating CLDN1. Conversely, another study conducted by Yang et al. (2020) demonstrated that miR-155 exerted tumor-promoting effect and significantly increased decitabine resistance by regulating TSPAN5 in triple-negative breast cancer. Some studies showed that miR-155 expression were significantly increased in tissues or blood of patients with cervical cancer and played pro-cancer role (Gocze et al., 2013; Okoye et al., 2019).

miR-155 has been also found to be play a pivotal role in multiple cancer models, including breast cancer, oral cancer, gastric cancer, liver cancer, and lung cancer. The underlying molecular mechanisms may be associated with the remodeling of tumor stroma, cellular viability, migration, invasion, cell cycle, metastasis, and immune escape. For example, miR-155 was suggested to serve as a biomarker for diagnostic and prognostic tool in breast cancer (Grimaldi et al., 2020). And the mechanisms for this action may be correlated to the activation of MMP, regulation of fibrillar collagen expression, and remodeling of the tumor stroma (Mushii et al., 2025). Liu et al. (2021) reported that miR-155 contributed to 5-fluorouracil resistance in oral squamous cell carcinoma by regulating the cell viability, migration, and invasion of the cancer cells. Exosomal miR-155 secreted by gastric cancer cells targeted C/EPBβ to inhibit adipogenesis and stimulate brown adipose differentiation, contributing to the development of tumorigenesis (Liu et al., 2022). miR-155 was also found to contribute to various processes such as the cell cycle, invasion, metastasis, and immune escape, thereby facilitating and exacerbating the progression of liver cancer (Xue et al., 2023). As reported, miR-155 has the ability to target PTEN and modulate signaling pathways like AP-1/NF-kB and AKT/ERK, exerting an impact on the tumor immune response in non-small cell lung cancer (NSCLC) (Wei et al., 2025). Therefore, miR-155 may play a crucial role in regulating several key biological processes of cancer development.

**FIGURE 1:**
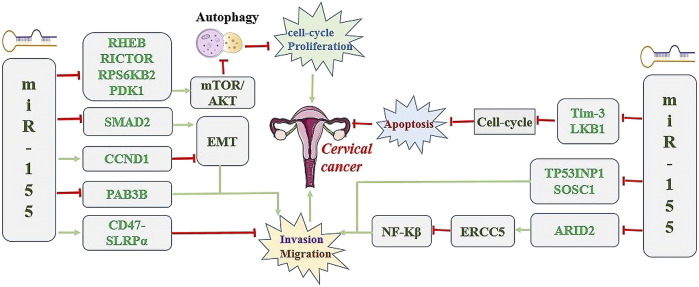
miR-155 biogenesis, function, and regulation diagram. Green Arrow indicates “activate”; Red Line indicates “inhibit”.

At present, a few additional studies found that the expression of miR-155 was elevated in cervical cancer samples, but significantly prolonged the survival time of patients with cervical cancer, indicating that miR-155 played a tumor suppressor role in cervical cancer (Yang et al., 2023; Paiva et al., 2015). Therefore, miR-155 may function as a tumor suppressor and protective factor in cervical cancer by modulating different target genes or signaling pathways. Figure 1 demonstrated the miR-155 biogenesis, function, and regulation diagram.

## The roles of miR-155 in the progress of cervical cancer

### miR-155 regulates cell cycle of cervical cancer

The cell cycle is a highly regulated process, controlling the growth, replication, and division of cells and consisting of distinct phases, including interphase (G1, S, and G2 phases) and mitotic phase (M phase) (Ahmed et al., 2023). Dysregulation of the cell cycle, a hallmark of cancer, contributes to uncontrolled cell proliferation and tumor development (Cheung et al., 2023). Cancer cells often exhibit aberrant cell cycle progression, characterized by accelerated proliferation, evasion of cell cycle checkpoints, and resistance to apoptosis (Yang H. et al., 2023). This dysregulation can result from mutations or alterations in key regulatory genes, such as tumor suppressor genes (e.g., p53, retinoblastoma protein) or oncogenes (e.g., cyclins, cyclin-dependent kinases) (Zabihi et al., 2023; Zhang et al., 2023). Additionally, cell cycle can be regulated by autophagy (Chen et al., 2023; Li et al., 2020). As we all known, mTOR activity negatively regulates autophagy. Li et al. (2023) reported that BML-275 significantly induced p21/cyclin D1/CDK4/6-mediated cell cycle G1/S arrest by promoting autophagy through inhibition of mTOR, which is beneficial for inhibition of prostate cancer progression. Furthermore, miR-15a/16 inhibited G1/S cell cycle transition by inducing autophagy through attenuating the phosphorylation of mTORC1, inhibiting the proliferation of human cervical carcinoma cells and enhancing the effects of chemotherapy medications (Huang et al., 2015). In addition, miR-155-5p was reported to suppress Wilms tumor by inactivating the PI3K/AKT/mTOR signaling pathway (Luo et al., 2020). Recently, Wan et al. (2014) demonstrated that overexpression of miR-155 attenuated the proliferation and cell cycle progression of cervical cancer cells. Furthermore, miR-155 was reported to induce autophagy by suppressing the phosphorylation of mTOR and AKT, as well as the protein levels of RICTOR, RHEB and RPS6KB2 (Wan et al., 2014). In addition, luciferase reporter indicated that miR-155 could directly interact with the 3′UTRs of RICTOR, RHEB and RPS6KB2 (Wan et al., 2014). However, the inhibition of miR-155 significantly inhibited the activation of autophagy by increasing the expression of RICTOR, RHEB and RPS6KB2, as well as the phosphorylation of mTOR (Wan et al., 2014). Importantly, miR-155 inhibition relived G1/S cell cycle arrest and promoted the proliferation of cervical cancer cells (Wan et al., 2014). Interestingly, autophagy inhibition relieved the inhibitory effect of miR-155 on G1/S cell cycle progression (Wan et al., 2014). These results suggest that miR-155 induces G1/S cell cycle arrest and inhibits cervical cancer cells proliferation by inducing autophagy via dysregulation of mTOR pathway.

In contrast, miR-155 can promote the progression of cervical cancer by regulating cell cycle. T cell immunoglobulin and mucin domain-containing protein 3 (Tim-3), presenting on the surface of macrophages, promotes tumor progression by accelerating macrophages transformation into foam cells through blocking secretion of NO and increasing the production of inflammatory factors by macrophages (Yang J. T. et al., 2020). In addition, Tim-3 also affects tumor growth by impacting cell cycle progression (Xiao et al., 2020). Yan et al. (2021) reported that the decreased expression of miR-155 induced cell cycle arrest in the S phases and increased apoptosis rate by activating Tim-3 signal pathway, which suppressed malignant characteristics of cervical cancer cells. miR-155 also play roles in normal cells and by regulating the cell cycle in normal tissues. For tumorigenesis, miR-155 is committed to participating in the cell cycle and immune escape and other processes to promote and intensify the development of cancer (Xue et al., 2023). Additionally, low expression of miR-155 induced cell cycle arrest and promoted apoptosis in cervical cancer cells (Lao et al., 2014). By contrast, miR-155 overexpression promoted the proliferation of cervical cancer cells (Lao et al., 2014). Mechanistic studies showed that miR-155 promoted the progression of cervical cancer through the inhibition of tumor suppressor LKB1 (Lao et al., 2014) Figure 2 shows the potential diagnostic prospects and therapeutic applications of miR-155 in cervical cancer.

**FIGURE 2:**
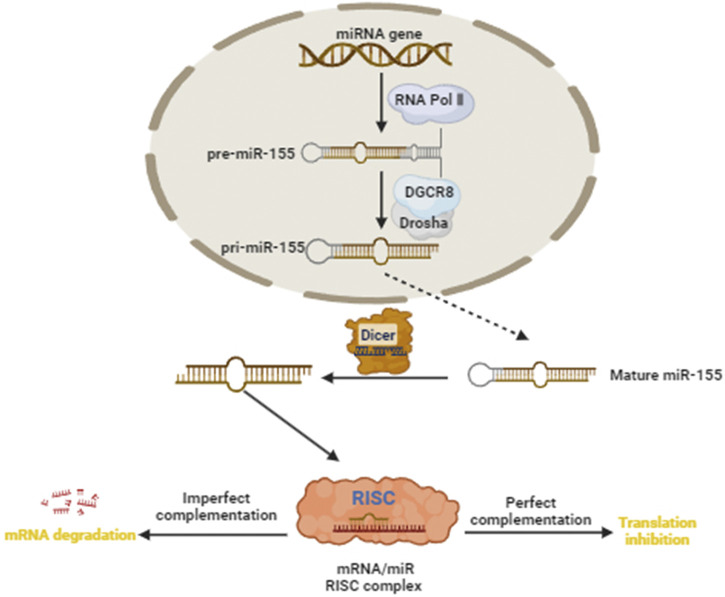
The underlying molecular mechanisms of miR-155 in the tumorigenesis and development of cervical cancer.

### miR-155 regulates invasion and metastasis of cervical cancer

Cell invasion and metastasis are critical processes in cancer progression (Adinew et al., 2022). Cell invasion involves cancer cells breaking through the extracellular matrix (ECM) and basement membrane surrounding the primary tumor, allowing them to invade adjacent tissues and blood or lymphatic vessels (Huang et al., 2023). Metastasis is a complex, multi-step process involving invasion, intravasation, circulation, extravasation, and colonization of distant organs (Huang et al., 2022). EMT has been reported to be a key process driving cancer cell invasion and metastasis (Jin et al., 2022). During EMT, epithelial cells lose their polarity and cell-cell adhesion properties, acquiring a mesenchymal phenotype with increased motility and invasiveness (Sabouni et al., 2023). EMT is regulated by various signaling pathways, including TP53INP1, TGF-β, Wnt, and Notch, as well as transcription factors such as Snail, Slug and Twist (Ang et al., 2023; Fan et al., 2023; Han et al., 2021; Liu A. et al., 2020). Studies have shown that miR-155 plays a role in promoting these processes. Kulkarni et al. (Kulkarni et al., 2021) reported that miR-155 induced cell migration and invasion of kidney cancer. In addition, miR-155 had also been shown to promote the migration and invasion of non-small-cell lung cancer (Li et al., 2021). Recent studies have shown that miR-155 significantly promoted the invasion and migration of cervical cancer cells, while the inhibition of miR-155 showed the opposite effect (Chen et al., 2019; Du et al., 2021). However, the specific mechanism of miR-155 on invasion and migration of cervical cancer cells were not investigated in these studies. The TP53 gene, one of the crucial cancer suppressors, responses to DNA damage, hypoxia and oncogene activation and is involved in the progression of various human tumors including breast carcinoma and colorectal cancer (Roche et al., 2023; Zhou et al., 2023). Additionally, TP53 has been reported to regulate malignant phenotypes of tumor cell through interacting with the tumor protein p53-induced nuclear protein 1 (TP53INP1) (Xia et al., 2019). TP53INP1, an important stress-response gene, is abnormally expressed in multiple tumors, including pancreatic carcinoma, breast cancer and liver cancer (Fang et al., 2019; Yu et al., 2018). Previous investigation suggested that miR-155 targeted TP53INP1 to facilitate the EMT process in liver cancer cells (Liu et al., 2015a; Liu et al., 2015b). Nevertheless, whether miR-155 regulates the invasion and metastasis of cervical cancer by modulating TP53INP1 is unclear. Recently, Li et al. (N. Li et al., 2019) demonstrated that expression of miR-155-5p increased, while expression of TP53INP1 decreased in cervical cancer tissues and cells compared with noncancerous cervical cancer tissues and cells. Furthermore, downregulation of miR-155-5p significantly suppressed migration and invasion of cervical carcinoma cell (Li N. et al., 2019). Also, tumor xenograft models demonstrated that the inhibition of miR-155-5p inhibited tumor growth *in vivo* (Li N. et al., 2019). In addition, overexpression of TP53INP1 significantly induced the apoptosis of cervical cancer cells and downregulation of TP53INP1 promoted the aggressiveness of cervical cancer cells (Li N. et al., 2019). Further study found that the level of TP53INP1 in cervical cancer cells was promoted by miR-155-5p inhibitor and downregulation of TP53INP1 reversed the inhibitory effect of miR-155-5p inhibitor on the proliferation and metastasis of cervical cancer cells (Li N. et al., 2019). These results suggested that miR-155-5p promoted cervical carcinoma cell invasion and metastasis by inhibiting the expression of TP53INP1. Consistent with this result, Zhang et al. (2018) reported that miR-155 promoted the progression of cervical cancer by inducing Th17/Treg imbalance and inhibiting SOSC1 expression. It has also been reported that miR-155-5p significantly promoted cervical carcinoma cell invasion and metastasis by regulating ARID2-ERCC5-NF-κB signaling pathway (Li H. et al., 2019). These findings indicate that miR-155 promotes cervical cancer cell invasion and metastasis, ultimately facilitating cervical cancer progression.

In contrast, some research has shown that miR-155 can act as an cancer-suppressor gene in cervical cancer by inhibiting invasion and metastasis of cancer cells. Autophagy, an important process for maintaining cell homeostasis, increases protein degradation and reduces protein synthesis and increases, thereby inhibiting tumor growth (Chen et al., 2022). The mTOR pathway plays an important role in autophagy regulation, and PDK1 has been considered as the activator of mTOR pathway (Sun et al., 2020). Qi et al. (2015) reported that PDK1-mTOR pathway inhibitor suppressed cell proliferation in resistant neuroblastoma. Wang et al. (2018) found that the expression of miR-155-5p was decreased in cervical cancer, whereas the level of autophagy was elevated. Furthermore, miR-155-5p downregulation inhibited autophagy, whereas miR-155-5p overexpression had the opposite effects in cervical cancer (Wang et al., 2018). In addition, miR-155-5p overexpression increased LC3 and decreased P62 protein expression in cervical cancer cells through suppressing PDK1/mTOR signaling, whereas miR-155-5p downregulation inhibited LC3 and recovered P62 protein expression by promoting the PDK1/mTOR pathway (Wang et al., 2018). These results indicated that miR-155-5p inhibited cervical cancer progression by enhancing autophagy through suppressing PDK1/mTOR signaling pathway. Additionally, miR-155-5p inhibited cervical cancer cell proliferation by downregulating RAB3B and upregulating CD47-SIRPa pathway (Huang et al., 2021; Xu et al., 2023; Yang T. J. et al., 2020). Moreover, miR-155 inhibited cervical cancer cell invasion and migration by repressing EMT through downregulating the expression of SMAD2 and CCND1 (Lei et al., 2012).

### miR-155 inhibits the responsiveness of cervical cancer cells to chemotherapeutic agents

The treatment landscape for ovarian cancer has evolved significantly, integrating chemotherapy, precision medicine, immunotherapy, novel drug conjugates, and surgical advancements to target diverse molecular profiles and clinical scenarios. Chemotherapy is considered a cornerstone in cancer treatment. Resistance to chemotherapeutic agents is involved in intrinsic and acquired resistance. Intrinsic resistance refers to the innate insensitivity of tumor cells to chemotherapy, often attributed to genetic alterations or molecular pathways that confer resistance (Al-Ansari et al., 2022). Acquired resistance, on the other hand, develops over time as tumors adapt to the cytotoxic effects of chemotherapy through mechanisms such as drug efflux, DNA repair, and alterations in drug targets (Antunes et al., 2022; Sharma et al., 2020). Thankfully, multiple studies have showed that miRNAs have crucial roles in the development and progression of chemotherapeutic resistance by affecting the intrinsic mechanisms (Palakurthi et al., 2012). Previous studies indicate that regulation of miRNAs can exert a drug-resistance-reversal effect (Bach et al., 2017; Santos and Almeida, 2020). Cisplatin is a commonly used chemotherapeutic agent for cervical cancer treatment (Himiniuc et al., 2022). Despite the initial clinical response, resistance often emerges after multiple courses of treatment. Evidences showed miR-155 plays an important role in this drug resistance process. Sayyed et al. (2021) reported that 155 inhibitor significantly reversed cisplatin resistance and inhibited tumor sphere formation in oral squamous cell carcinoma. Since miR-155 was found to be involved in resistance to chemotherapy in cancer (Palakurthi et al., 2012), miR-155 inhibitors might be effective on chemotherapeutic resistance in cervical cancer. It was observed that cisplatin treatment invoked the increased expression of miR-155 and the decreased expression of caspase-3 in cervical cancer cells in a time-dependent manner (Zhao et al., 2020). Overexpression of miR-155 led to a decreased apoptosis rate of cervical cancer cells under Cisplatin treatment (Zhao et al., 2020). However, caspase-3 overexpression played the opposite role (Zhao et al., 2020). Further studies revealed that miR-155 overexpression reversed the effects of caspase-3 overexpression (Zhao et al., 2020). These results suggested that miR-155 provides cervical cancer cells with anti-apoptotic abilities by inhibiting the expression of caspase-3, leading to cisplatin resistance. However, hitherto, only one previous report concerning the correlation between miR-155 and drug resistance in cervical cancer. Therefore, more studies are needed to fully investigate the miR-155 in cisplatin resistance in cervical cancer.

### Potential of miR-155 as a biomarker in cervical cancer

As summarized in Table 1, the expression level of miR-155 in cervical cancer tissues is regulated by several biological processes. Thus, miR-155 might be considered as a biomarker in cervical cancer. However, many obstacles, including tissue specimens, invasiveness and cost, limiting this approach. Interestingly, a growing number of studies suggested that the circulating level of miR-155 is associated with tumorigenesis, and it could be easily measured with molecular biology assays. Since microRNA can also be actively secreted by cells and are extremely stable in plasma, they can be frequently used as a novel class of disease biomarkers (Favero et al., 2021). The properties of miR-155 as biomarker have been studied in cervical cancer. The expression of miR-155-5p was significantly elevated in cervical cancer tissues (Jihad and Issa, 2021). Moreover, The expression levels of miR-155 is strikingly higher in patients with low-grade squamous intraepithelial lesions and high-grade squamous intraepithelial lesions than in the healthy individuals (Azimi et al., 2021). Although, the levels of miR-155-5p showed no statistical differences between patients with low-grade squamous intraepithelial lesions and high-grade squamous intraepithelial lesions (Azimi et al., 2021). In addition, overexpression of miR-155 was sufficient to discriminate with cervical cancer tissues from normal (Park et al., 2017). Further analysis found that the expression level of miR-155-5p also increased in the urine and serum of patients with cervical cancer compared to healthy controls (Aftab et al., 2021). These studies have demonstrated the sensitivity and specificity of miR-155 in distinguishing cervical cancer from benign lesions or healthy controls, highlighting its potential as a non-invasive biomarker for early detection and screening programs. Furthermore, the prognostic significance of miR-155 in cervical cancer has been extensively investigated. It was reported that increased miR-155-5p expression level was significantly associated with TNM clinical stage, lymph nodes metastasis, vascular invasion and poor prognosis in cervical cancer patients, indicating miR-155 may serve as an unfavorable factor for survival (Aftab et al., 2021; Fang et al., 2016). Conversely, another study showed that low expression of miR-155 in cervical cancer patients is linked to poor overall survival compared to those with high miR-155 expression (Santos et al., 2023). Furthermore, high expression of miR-155 was also associated with improved survival rates for clinical stages I and II, indicating miR-155 may serve as a favor prognostic factor for cervical cancer patients (Santos et al., 2023). In conclusion, miR-155 may serve as a prognostic biomarker for cervical cancer population, despite the unconformable results.

**TABLE 1:** Characteristics of the relevant clinical studies reported the expression of miR-155 in cervical cancer.

Study/ References	Expression	Role of miR-155	Clinical findings
Wang et al., 2016	Up	tumor promoter	miR-155 is upregulated in the cervical cancer tissues as compared with normal tissues, indicating an oncogenic role in cervical cancer
Gocze et al. (2013)	Up	tumor promoter	Overexpression of miR-155 (p = 0.021) is observed in human cervical cancer samples
Okoye et al. (2019)	Up	tumor promoter	Higher expression of miR-155 is observed in sera of cervical cancer patients than in sera of normal patients
Chen Y. et al. (2021)	Down	tumor inhibitor	Although the expression level of miR-155 is higher in patients with cervical cancer, miR-155 significantly prolonged survival time, thus, indicating its anti-cancer function in patients with cervical cancer
Jihad and Issa (2021)	Up	tumor promoter	miR-155 is statistically significant upregulated in cervical cancer tissues as compared to controls group
Azimi et al. (2021)	Up	tumor promoter	miR-155-5p is upregulated in LSIL and HSIL and can be served as a prognostic biomarker for cervical cancer patients
Park et al. (2017)	Up	tumor promoter	miR-155 is significantly upregulated in cervical cancer tissues compared to normal cervical tissues
Aftab et al. (2021)	Up	tumor promoter	miR-155-5p expression in non-invasive urine samples serves as a biomarker for early diagnosis and prognosis of cervical cancer
Fang et al. (2016)	Up	tumor promoter	miR-155 expression is increased in cervical cancer tissues and correlated with lymph nodes metastasis, FIGO stage and vascular invasion
Santos et al. (2023)	Up	tumor promoter	Low miR-155 expression level in cervical cancer is associated with poor overall survival

### MiRNA, a new treatment strategy for cervical cancer

Table 2 showed the characteristics of the relevant experimental studies reported the role of miR-155 in cervical cancer. It has been shown that Cancer-associated miRNAs can be generally classified as oncogenic miRNAs or tumor suppressor miRNAs. A miRNA can assume the role of a tumor suppressor gene when it targets an oncogene, conversely, it can assume the role of an oncogene, if it’s critical target is a tumor suppressor gene. Notably, a single miRNA can target multiple mRNA of a great variety of genes responsible for cancerous processes, leading to a dual role as tumor suppressor as well as oncogenic function, depending on the specific cancer type (Qie and Sang, 2022; Yang S. et al., 2023). Due to these properties, targeting dysregulated miRNAs holds immense therapeutic potential for modulating cancer behavior and progression. miR-155 dysregulation alters critical mRNA networks, driving cervical cancer pathogenesis. Validated targets (e.g., RHEB, RICTOR, TP53INP1, SOSC1, PDK1, and SMAD2) offer promising therapeutic entry points, while unresolved axes (e.g., miR-155) warrant further study.

**TABLE 2:** Characteristics of the relevant experimental studies reported the role of miR-155 in cervical cancer.

Study/ References	Expression	Role	Target	Associated genes/pathways	Main findings
Paiva et al. (2015)	Down	tumor inhibitor	NA	NA	miR-155 protects against HPV16- induced carcinogenesis
Chen Y. et al. (2021)	Down	tumor inhibitor	NA	NA	Although the expression level of miR-155 is higher in patients with cervical cancer, miR-155 significantly prolonged survival time, thus, indicating its anti-cancer function in patients with cervical cancer
Wan et al. (2014)	Down	tumor inhibitor	RHEB RICTOR, and RPS6KB2	NA	miR-155 attenuates cervical cancer cell proliferation and cell cycle progression by inducing autophagy through directly interacting with the 3′UTRs of RHEB, RICTOR, and RPS6KB2
Yan et al. (2021)	Up	tumor promoter	NA	NA	Composite nanoparticles suppress malignant characteristics of CC cells by promoting secretion of macrophage inflammatory factors through inhibiting miR-155 on the surface of macrophages
Lao et al. (2014)	Up	tumor promoter	LKB1	NA	miR-155 promotes cervical cancer progression by inhibiting LKB1 expression
Du et al. (2021)	Up	tumor promoter	NA	NA	Propofol inhibits the growth and invasion of cervical cancer cells via suppressing miR-155 expression
Chen et al. (2019)	Up	tumor promoter	NA	TCF-7/miR-155	TCF-7 inhibits the migration and invasion of cervical cancer cells by suppressing miR-155
Li N. et al., 2019	Up	tumor promoter	TP53INP1	NA	miR-155-5p promotes the development of cervical cancer cell by inhibiting the expression of TP53INP1
Zhang et al. (2018)	Up	tumor promoter	SOCS1	NA	miR-155 contributes to the development of cervical cancer by suppressing the expression of SOSC1 and inducing Th17/Treg imbalance
Li H. et al. (2019)	Up	tumor promoter	ARID2/ERCC5/NF-κB	NA	miR-155-5p promotes cervical cancer cellular proliferation, migration and invasion by regulating ARID2-ERCC5-NF-κB signaling pathway
Wang et al. (2018)	Down	tumor inhibitor	PDK1/ mTOR	NA	miR-155-5p inhibits malignant cervical change by enhancing autophagy through suppressing PDK1/mTOR signaling
Yang L. W. et al. (2020)	Down	tumor inhibitor	NA	LncRNA UCA1/miR-155	LncRNA UCA1 promotes cervical cancer cell proliferation, migration and invasion by suppressing miR-155 expression
Huang et al. (2021)	Down	tumor inhibitor	CD47-SIRPα	NA	Vitamin E succinate exerts an antitumor activity by inhibiting CD47-SIRPα pathway through upregulating miR-155 expression
Xu et al. (2023)	Down	tumor inhibitor	RAB3B	circ_0000337/miR-155-5p	circ_0000337 promotes cervical cancer cell proliferation by upregulating RAB3B through suppressing miR-155-5p
Lei et al. (2012)	Down	tumor inhibitor	TP53, SMAD2, and CCND1	NA	miR-155 reversed EGF-induced EMT by upregulating TP53, downregulating SMAD2 expression levels, and restraining cell growth by inhibiting CCND1 expression
Zhao et al. (2020)	Up	tumor promoter	Caspase-3	miR-503/miR-155	miR-503 suppresses the drug resistance of CSCC cells by upregulating Caspase-3 expression through inhibiting miR-155

One of the key strategies for miRNA-based therapy in cervical cancer involves the use of synthetic oligonucleotides, such as miRNA mimics or inhibitors, to modulate the expression of specific miRNAs. Delivering miRNA mimics to restore the function of tumor-suppressive miRNAs or administering miRNA inhibitors to block oncogenic miRNAs offers a targeted approach to attenuate cancer progression and enhance treatment efficacy (Yang S. C. et al., 2022). Nanoparticle-based delivery systems and viral vectors have been explored for efficient and targeted delivery of miRNA therapeutics to cancer cells (Kara et al., 2022; Liang et al., 2022).

Some oncogenic miRNAs are often overexpression in cancers, prompting the use of miRNA inhibition as replacement therapy. The inhibition of miRNA reduces or potentially eliminate their harmful activity. On the other hand, tumor-suppressive miRNAs are typically downregulated in cancers, leading to the development of miRNA-based replacement therapy. These therapies aim to deliver miRNAs to patients in order to modulate abnormal cellular functions. To date, various miRNAs have showed significant therapeutic potential in cervical cancer. For example, miR-145 is downregulated in cervical cancer specimens and has the ability to suppress multiple genes involved in tumor progression, such as Sox2, Nanog and Oct4 (Azizmohammadi et al., 2017; Zhou et al., 2017). Study has shown that miR-145 overexpression significantly inhibited the proliferation, migration, and invasion of cervical cancer cells (Hu et al., 2023). In addition, injection of adenovirus-miR-145 significantly suppressed tumor growth in nude mice (Zhou et al., 2017). Similarly, miR-145 mimics significantly inhibits the growth of xenograft tumor and prolongs the survival time of mice (Hu et al., 2023). Inhibition of oncogenic miRNAs also potentially be used for lung cancer therapy. For instance, circular RNA CDK6 competitively inhibits miR-449a and regulates EMT process, leading to the inhibition of cervical cancer proliferation and metastasis (Zhong et al., 2022).

**FIGURE 3:**
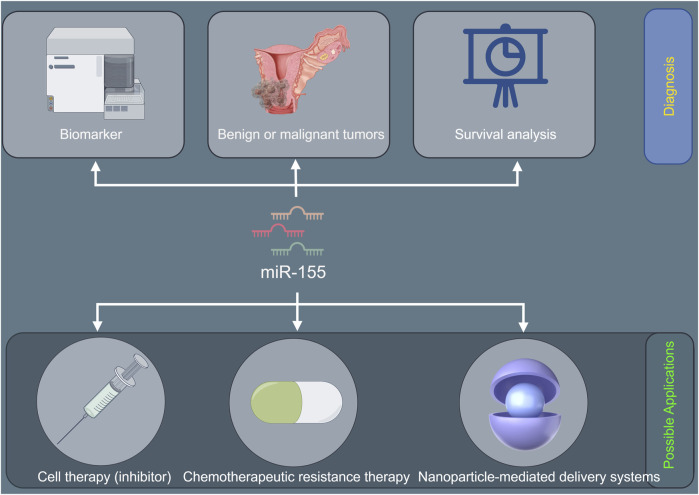
The potential diagnostic prospects and therapeutic applications of miR-155 in cervical cancer.

Recently, miR-155 has raised significant concerns due to its therapeutic potential in cervical cancer. It is reported that knocking down miR-155 by RNA interference promotes the proliferation of cervical cancer cells *in vitro* (Huang et al., 2021). Some therapeutic drugs, like Vitamin E succinate, have been found to upregulate miR-155 expression, leading to inhibition of cervical cancer cells and tumor growth and volume both *in vitro* and *in vivo* (Huang et al., 2021). Further study have revealed that Vitamin E succinate upregulates miR-155 expression, which regulates CD47-SIRPα pathway, ultimately suppressing cervical cancer cell growth and tumor progression (Huang et al., 2021). In contrast, the inhibition of miR-155 also suppress the progress of cervical cancer. Yan et al. (Yan et al., 2021) reported that chitosan nanoparticles (NPs) loaded with TGF-β could treat cervical cancer through regulation of miR-155. The composite NPs was able to effectively enter cervical cancer cell, leading to downregulation of miR-155, which promotes secretion of macrophage inflammatory factors and regulates cell apoptosis and cycle, ultimately suppressing malignant characteristics of cervical cancer cells (Yan et al., 2021). Another study also demonstrated that miR-155 mimic significantly promotes the migration and invasion of cervical cancer cells (Chen et al., 2019). Importantly, inhibition of miR-155 by LncRNA TCF7 inhibits the migration and invasion of cervical cancer cells (Chen et al., 2019). Furthermore, inhibition of miR-155 by LncRNA TCF7 can reduce tumor size in cervical cancer xenograft models (Chen et al., 2019). These findings highlight the potential of microRNA-based cancer therapy, providing an opportunity to disrupt multiple cancer-relevant processes, particularly in cervical cancer therapy. Figure 3 displayed the underlying molecular mechanisms of miR-155 in the tumorigenesis and development of cervical cancer.

## Limitations and perspectives

The oncogenic role of miR-155 in cervical cancer likely involves HPV interaction, immune dysregulation, and epigenetic remodeling. Prioritizing studies on its crosstalk with HPV oncoproteins, inflammatory pathways, and immune checkpoints will address critical gaps. However, there is a lack of in-depth exploration into the dynamic changes of miR-155 expression during the multi-step process of cervical carcinogenesis. Longitudinal studies tracking miR-155 levels from the initial HPV-associated cellular changes to the development of invasive cancer are scarce. Understanding these temporal changes could provide insights into the critical time points when miR-155 exerts its oncogenic effects, potentially leading to more targeted preventive strategies. Therapeutically, combining miR-155 inhibition with immunotherapy or epigenetic drugs offers a promising multi-target strategy. For example, anti-miR-155 oligonucleotides may be designed to specifically bind to miR-155, blocking its function. *In vitro* studies have already demonstrated that knockdown of miR-155 inhibits cell proliferation, migration, and invasion of cervical cancer cells. However, the challenge lies in delivering these anti-miR-155 oligonucleotides effectively to the tumor site *in vivo*. Nanoparticle-mediated delivery systems could be a promising solution. These nanoparticles can encapsulate the anti-miR-155 molecules, protecting them from degradation and facilitating their uptake by cancer cells. In future, validation of miR-155 as a biomarker could further personalize cervical cancer management. Figure 3 shows the potential diagnostic prospects and therapeutic applications of miR-155 in cervical cancer.

## Conclusion

The present review comprehensively investigated the relationship between miR-155 and cervical cancer. The majority of the studies reveal that miR-155 plays a pro-cancer role in cervical cancer, with high expression levels associated with poor prognosis. Mechanistically, miR-155 promotes cervical cancer progression by regulating critical steps in cancer development, such as cell cycle, migration and invasion. In addition, miR-155 is associated with drug resistance in cervical cancer, with studies showing that the inhibition of miR-155 can promote drug sensitivity and potentially reverse resistance. In summary, these findings demonstrate that miR-155 may function as a valuable biomarker and therapeutic target for cervical cancer. However, since miR-155 can regulate different targets, miR-155 functions as a tumor suppressor in cervical cancer, with factors such as cell type, stage, and tumor microenvironment influencing its effects. Further studies are still needed to determine the clinical application as many aspects remain unresolved.
